# Transcriptional Dynamics of Grain Development in Barley (*Hordeum vulgare* L.)

**DOI:** 10.3390/ijms20040962

**Published:** 2019-02-22

**Authors:** Jianxin Bian, Pingchuan Deng, Haoshuang Zhan, Xiaotong Wu, Mutthanthirige D. L. C. Nishantha, Zhaogui Yan, Xianghong Du, Xiaojun Nie, Weining Song

**Affiliations:** 1 State Key Laboratory of Crop Stress Biology in Arid Areas, College of Agronomy and Yangling Branch of China Wheat Improvement Center, Northwest A&F University, Yangling, Shaanxi 712100, China; brian1791@nwafu.edu.cn (J.B.); dengpingchuan@nwsuaf.edu.cn (P.D.); zhanhaoshuang@nwsuaf.edu.cn (H.Z.); wuxiaotong@nwsuaf.edu.cn (X.W.); mdlcnishantha@gmail.com (M.D.L.C.N.); xianghongdu@nwsuaf.edu.cn (X.D.); 2 Huazhong Agricultural University, Wuhan 430070, China; gyan@mail.hzau.edu.cn; 3 Joint Research Center for Agriculture Research in Arid Areas, Yangling, Shaanxi 712100, China

**Keywords:** Barley, Grain development, Transcriptional dynamics, RNA editing, RNA-seq

## Abstract

Grain development, as a vital process in the crop’s life cycle, is crucial for determining crop quality and yield. However, the molecular basis and regulatory network of barley grain development is not well understood at present. Here, we investigated the transcriptional dynamics of barley grain development through RNA sequencing at four developmental phases, including early prestorage phase (3 days post anthesis (DPA)), late prestorage or transition phase (8 DPA), early storage phase (13 DPA), and levels off stages (18 DPA). Transcriptome profiling found that pronounced shifts occurred in the abundance of transcripts involved in both primary and secondary metabolism during grain development. The transcripts’ activity was decreased during maturation while the largest divergence was observed between the transitions from prestorage phase to storage phase, which coincided with the physiological changes. Furthermore, the transcription factors, hormone signal transduction-related as well as sugar-metabolism-related genes, were found to play a crucial role in barley grain development. Finally, 4771 RNA editing events were identified in these four development stages, and most of the RNA editing genes were preferentially expressed at the prestore stage rather than in the store stage, which was significantly enriched in “essential” genes and plant hormone signal transduction pathway. These results suggested that RNA editing might act as a ‘regulator’ to control grain development. This study systematically dissected the gene expression atlas of barley grain development through transcriptome analysis, which not only provided the potential targets for further functional studies, but also provided insights into the dynamics of gene regulation underlying grain development in barley and beyond.

## 1. Introduction

Grain development is one of the most essential processes for the life cycle of plants, and especially for crops, which is not only critical for colonization of the environment for plant survival, but also provides the main food source for human beings [1,2,3,4]. It is a very complex biological process involving multiple metabolic regulation pathways, which can be further divided into two major phases, embryogenesis and maturation. During the embryogenesis process, embryonic cells divide and go through characteristic stages of development, while maturation mainly involves accumulation of organic materials such as protein, lipids, and carbohydrates [4,5,6,7]. A better understanding of the molecular mechanisms of grain development will contribute to crop improvement, including seed formation, morphogenesis, and storage reserve accumulation. During the last few years, a larger number of studies have been conducted to investigate the specific genes or pathways controlling grain development in different species, particularly in model organisms such as rice [8] and Arabidopsis [3,5,9]. These studies have provided important information on both of the regulators of transcription and the genes involved in grain development.

Barley (*Hordeum vulgare*), a member of the Poaceae family, was domesticated thousands of years ago and has been utilized mainly for animal feed, malting, and the brewing industry, and as a human food source [1,10]. Because of its diploid nature and rich genetic diversity, barley is also a well-studied crop regardless of genetics or genomics and once acted as a model plant for Triticeae research. Since the grain is the agriculturally most important organ of crops, many studies have been carried out on barley grain development [1,6,7,11,12,13]. Preliminary transcriptomic analyses of barley seeds have provided important information regarding tissue-specific metabolic pathways and the regulatory network controlling grain development, including storage reserve accumulation [6], photosynthesis [7], hormone biosynthesis [6,14], programmed cell death [12], sucrose transport [13], and zinc trafficking [15]. Particularly, a comparative study of transcript and metabolite profiling at both temporal and spatial levels dissected that glumes acted as important transitory resource buffers during barley endosperm filling [16]. Furthermore, the differentially expressed genes (DEGs) involved in barley grain development through RNA-seq are also reported and available in barleyGenes—Barley RNA-seq Database (https://ics.hutton.ac.uk/barleyGenes/about_material.html). However, the transcriptomic dynamics of barley grain development are not well understood, and in particular, there is little known about the regulatory network at present. Recently, the high-quality reference sequence of the barley genome was completed, which provides the unprecedented opportunity to investigate the dynamic of the barley grain development at the whole transcriptome level [17]. Here, we performed a study of mRNA-seq analysis at four stages spanning important developmental phases of seed filling with the purpose to the transcriptional dynamics and regulatory process of grain development in barley.

## 2. Results

### 2.1. RNA-Seq Analysis of Grain Development in Barley

In this study, the dynamics of mRNA abundance were systematically investigated at four important stages (3, 8, 13, and 18 DPA) of grain development in barley (Figure S1). Totally, 52.69 Gb of raw data was obtained for all of the samples, with the average of about 65 million pair-end reads with 100 bp in size for each sample (Table S1). After removing adaptor sequences and low-quality reads (reads with N and empty reads), there were around 521 million clean reads remaining, which represented 98.97% of the raw data. After quality filter, all of the clean reads were mapped to the barley reference genome [17]. Results showed that on average, 55.10 million paired-end reads (84.53%) could be uniquely mapped to the reference genome [17], with the range from 53.86 (82.75%) to 56.28 million reads (86.29%) (Figure S2A). Out of 39,734 annotated protein-coding genes in the barley genome, 19,165 (48.23%) genes were found to be expressed during grain development (Table S1), which was consistent with a previous study in Arabidopsis (~44%) and hexaploid wheat (~55%) [2,18].

**Figure 1:**
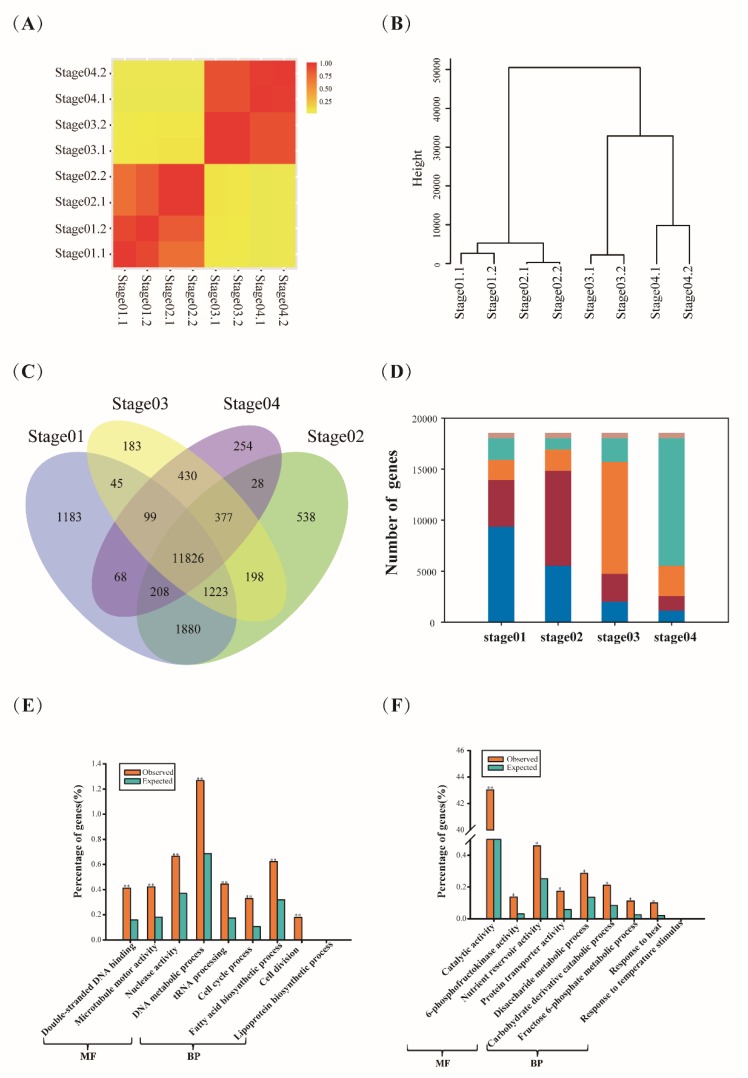
Global characterization of gene expression patterns during the four stages of barley grain development. (**A**) Correlation coefficients between gene expression data sets from two biological duplicates and stages. (**B**) Cluster dendrogram showing global relationships of gene expression in different stages. The branch length indicates the degree of variance. (**C**) Venn diagram analyses of stage-specific genes in barley. (**D**) Overview of genes with different activity degree among the four stages. The different number suggests different activity ranking at each stage and the activity degree decreases as the number increases. For example, ‘1′ indicates gene showed the most activity at this stage and ‘4′ suggests the most inactive stage. (**E**) to (**F**) Functional categories of genes showing peak expression at the prestorage phase (stage01 and stage02) (**E**) and storage phase (stage03 and stage04). (**F**) Padj-adjusted *p* values, * *p* < 0.05 and ** *p* < 0.01. Observed, numbers of genes observed in this study; Expected, numbers of genes in this same category of annotated barley gene models.

To determine whether this gene expression correlated with developmental stages, reads numbers were firstly normalized to RPKM value, and then were subjected to the usual correlation coefficient (*R*^2^) and hierarchical clustering analysis [19]. We found that two biological replicates of all samples showed consistent determinations of transcript abundance with a coefficient (*R*^2^) greater than 0.93 (Figure 1A). In addition, gene expression correlations between different stages were compared. Results found that the coefficients of 3 DPA (stage01) and 8 DPA (stage02) were 0.73 (*R*^2^ > 0.73) and those of 13 DPA (stage03) and 18 DPA (stage04) were higher with the value of 0.89. However, the coefficient between 13 DPA (stage03) and 8 DPA (stage02) was only 0.10, indicating a large change in gene expression pattern occurred during the process. It may be the transition from prestorage phase to storage phase, which was consistent with a previous study finding that the most dramatic transcriptional and physiological changes occurred during transition from the prestorage to the storage phase [6]. Similar stage expression patterns were supported by correlation dendrogram analysis (Figure 1B). The correlation dendrogram demonstrated that the four stages were clearly clustered into two groups, namely 3 DPA (stage01) and 8 DPA (stage02) grouped together, and 13 DPA (Stage03) and 18 DPA (Stage04) clustered into another group. Then, we also used the publicly available barley grain RNA-seq data (5 DPA and 15 DPA) to perform correlation analysis. Results found that 3 DPA and 8 DPA showed high correlation coefficient with 5 DPA and 13 DPA and 18 DPA showed high correlation coefficient with 15 DPA, which also could be clustered into two groups (Figure S3). Overall, the correlation analyses results were in agreement with the developmental order as expected, which were consistent with the previous study [7].

Furthermore, we compared the changes of gene expression abundance at different development stages. The total number of expressed genes in each sample varied from 13,290 (stage04) to 16,532 (stage01) (Table S2) and approximately 63.79% (11,826/18,540) of them were expressed across all of these four stages (Figure 1C). Interestingly, the majority of the genes (80.35%) exhibited peak expression at the prestorage phase (stage01 and stage02) rather than the storage phase (stage03 and stage04) (Figure 1D). GO (Gene ontology)analysis identified the peak expressed genes were significantly enriched in molecular functions at each stage. For example, at the prestorage phase, the peak genes were enriched in terms of cell cycle, DNA, RNA, peptidase biosynthesis, and modification such as cell cycle process (GO: 0022402), cell division (GO:0051301), nuclease activity (GO:0004518), DNA metabolic process (GO: 0006259), tRNA processing (GO: 0008033), double-stranded DNA binding (GO:0003690), microtubule motor activity(GO:0003777), lipoprotein biosynthetic process (GO:0042158), fatty acid biosynthetic process (GO:0006633) (Figure 1E) (Table S3). However, at the storage phase, gene sets were enriched in terms of matter accumulation, such as nutrient reservoir activity (GO: 0045735), catalytic activity (GO: 0003824), 6-phosphofructokinase activity (GO: 0003872), fructose 6-phosphate metabolic process (GO: 0006002), response to temperature stimulus (GO:0009266), response to heat(GO:0009408), disaccharide metabolic process (GO:0005984), protein transporter activity (GO:0008565), carbohydrate derivative catabolic process (GO:1901136) (Figure 1F) (Table S4).

Genes displaying enriched expression patterns in a given developmental stage or tissue are important for understanding the specialized processes within these stages or tissues. As described previously, an empirical cutoff value for positively expressed genes (FPKM ≥ 1) was used to detect stage-specific expression candidates [20]. In our study, we identified 2158 stage-enriched genes, of which stage01 possessed the highest number (1183), followed by stage02 (538), stage04 (254), and stage03 (183) (Figure 1C). GO enrichment results showed the stage-specific expression genes involved in DNA metabolic process (GO:0006259), DNA repair (GO:0006281), reproductive process (GO:0022414), RNA processing (GO:0006396) for stage01, transport (GO:0006810), single-organism transport (GO:0044765), lipid metabolic process (GO:0006629), carbohydrate binding (GO:0030246) for stage02, and aromatic compound biosynthetic process (GO:0019438), organic cyclic compound biosynthetic process (GO:1901362), biosynthetic process (GO:0009058), organic substance biosynthetic process (GO:1901576) for stage03, as well as serine-type endopeptidase inhibitor activity (GO:0004867), transferase activity, transferring hexosyl groups (GO:0016758) for stage04 (Table S4), which represented the grain developmental program and biological process.

### 2.2. Transcriptome Dynamics of Grain Development in Barley

**Figure 2:**
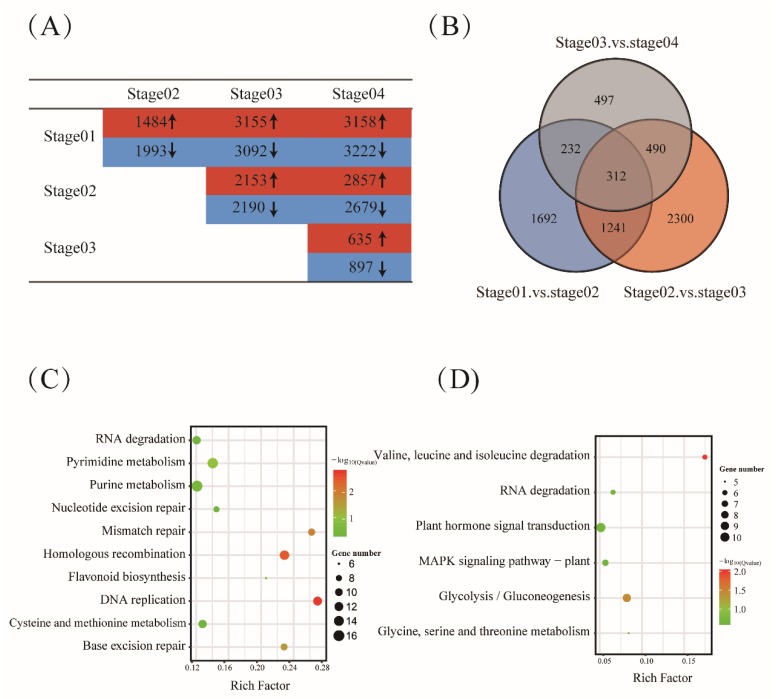
Differential expression genes during barley grain development. (**A**) Number of genes showing up- or downregulated expression during barley grain development (Padj < 0.05 and |log2Ratio| ≥1). (**B**) Venn diagram analyses of genes that were differentially expressed between any two consecutive stages in barley. (**C**) KEGG enrichment analysis of DEGs in cluster V during barley grain development. (**D**) KEGG enrichment analysis of DEGs in cluster VII.

To capture temporal changes during barley grain development, we compared the gene expression levels between these four stages. Using the Padj < 0.05 and |log2Ratio| ≥1 as threshold, approximately 51.38% of the expressed genes (9527/18,541) were identified as significantly differential expressions, with values ranging from 1532 (stage04 vs. stage03) to 6380 (stage 04 vs. stage01) (Figure 2A, Figure S4, Table S5). When comparing adjacent time points (for example, stage02 vs. stage01 and stage03 vs. stage02), we found that the largest variation in differentially expressed genes occurred between stage03 and stage02, which was consistent with the original correlation analyses. In addition, obvious variations were found on the composition of the differentially expressed genes between any two adjacent time points (Figure 2B). A total of 312 genes were significant differentially expressed at all of the four developmental stages, suggesting the specialized functions and differentiation during barley grain development that might be involved in different sets of genes with distinct patterns of expression. All the differentially expressed genes were further used to define clusters displaying distinct temporal patterns during development. A K-mean clustering method was conducted with the squared Euclidean distance measure, and all the genes potentially involved in the regulation of grain filling were classified into nine categories (Figure S5 and Table S6) determined using the Calinski–Harabasz (CH) index.

Based on the growth characteristics, starch accumulation patterns, and metabolite profiles, the major events of barley endosperm development were classified into four main stages: prestorage phase (0–5 DPA), late prestorage or transition phase (6–10 DPA), early storage phase (11–15 DPA), and levels off stages (16–20 DPA) [6]. The first four clusters (I–IV) of differentially expressed genes were preferentially expressed at these four development stages, respectively (Table S6). Cluster I comprised 1823 genes with the peak expression at the first stage and then declined sharply at later stages. This cluster showed a clear enrichment of genes involved in protein, chromosome, and cell wall pathways such as protein dimerization activity (GO:0046983), chromosome organization (GO:0051276), cell wall organization (GO:0071555). The transition phase is characterized mainly by an initial accumulation of starch. Expecting for some pathways involved in preparing for starch accumulation such as protein and RNA, genes preferentially expressed at this phase (cluster II, 1311 genes) were also enrichment for some carbohydrate metabolism pathways such as photosynthesis (GO:0015979), transporter activity (GO:0005215), and carbohydrate binding (GO:0030246). Expression of genes in cluster III group (472 genes) activated significant enrichment of genes involved in intracellular transport (GO:0046907), macromolecule localization (GO:0033036), phosphorelay signal transduction system (GO:0000160), pyrophosphatase activity (GO:0016462), and nutrient reservoir activity (GO:0045735). At the levels off stage, there was a significant increase in expression (cluster IV, 746 genes) of genes related to multicellular organism development (GO:0007275) and developmental process (GO:0032502). Cluster V group contained the largest number of differentially expressed genes (2,217 genes), which showed an increased expression at the prestorage phase (stage01 and stage02). In preparation for seed filling, genes related to protein, RNA, cell organization, and cell division were activated and significantly enriched for cell cycle (GO:0007049), cytokinesis (GO:0000910), lipid metabolic process (GO:0006629), cellulose synthase activity (GO:0016759), RNA processing (GO:0006396), nucleosome organization (GO:0034728), chromosome organization (GO:0051276), cellular component biogenesis (GO:004085), protein metabolic process (GO:0019538), organic acid metabolic process (GO:0006082). The KEGG pathway analysis found they were significantly enriched in the DNA replication, mismatch repair pathway (Figure 2C). Transcripts belonging to cluster VII with a peak of expression at the storage phases (stage03 and stage04) were mainly involved in nutrient reservoir activity (GO:0045735), enzyme inhibitor activity (GO:0004857), peptidase inhibitor activity (GO:0030414). Compared to cluster V, genes in this cluster were significantly enriched in glycolysis/gluconeogenesis and plant hormone signal transduction pathway (Figure 2D). Other functional classes, such as stress, transport, cell wall, protein, and RNA, were also over-represented in cluster VI, cluster VIII, and cluster IX (Table S6).

### 2.3. Functional Analyses of Differentially Expressed Transcription Factors during Barley Grain Development

**Figure 3:**
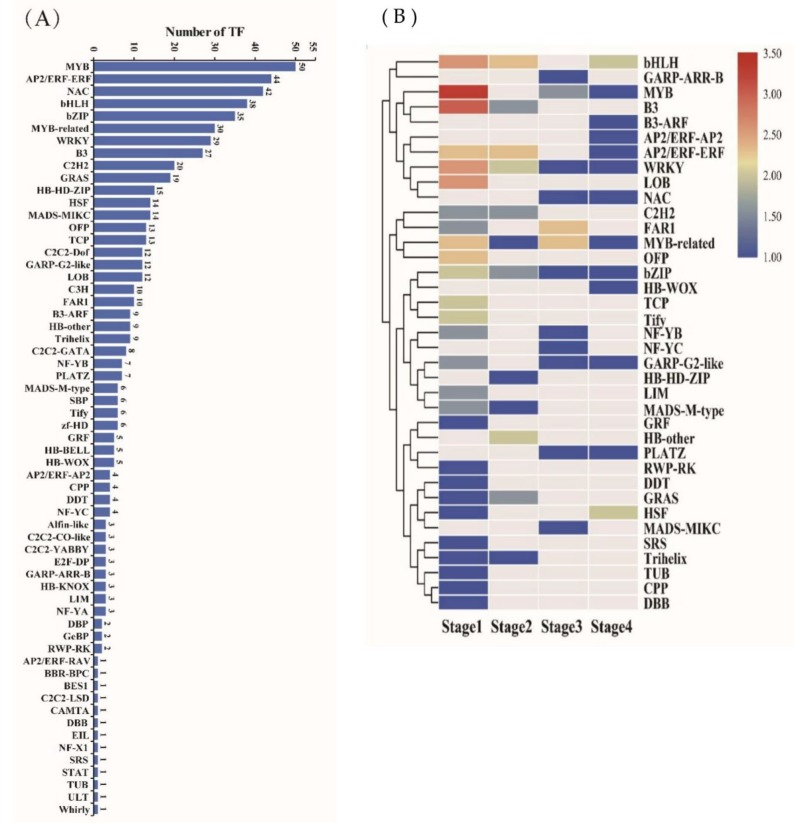
Characterization of transcription factors (TFs) differentially expressed during barley grain development. (**A**) Number of transcription factors (TFs) differentially expressed between any two stages of barley grain development. (**B**) Characterization of TF in each expression pattern.

Transcription factors (TFs) are one of the main factors for signal transduction and regulating gene expression, which play important roles in controlling the growth, development, and stress response in plants. We further identified the transcription factor gene among the identified differentially expressed genes. Results found that there were 606 transcription factors in these DEGs, which could be assigned into 61 families based on the presence of conserved domains. Among them, MYB family members were the most prominent (50), followed by AP2/ERF-ERF (44), NAC (42), bHLH (38), bZIP (35), and MYB-related (30) (Figure 3A and Table S7). MYB proteins are key factors in regulatory networks controlling development and metabolism under biotic and abiotic stress [21]. In this dataset, 17 (34%) MYB TFs members abundantly expressed at the prestorage phase, while another 10 genes (20%) were preferentially expressed at the storage phase (Figure 3B). The differential function of MYBs contributed to controlling development and stress response, which might be the reason the MYB family holds the largest TFs family during grain development. In addition, MADS transcription factor family, known to play crucial roles in flower organ development [22], were found to be abundantly expressed (20 members), implying an unexplored distinct role of MADS box TFs during barley grain development. On the other hand, only 22 TFs (36.73%) were expressed at the level of FPKM ≥50, indicating that most TFs were not highly expressed in grain (Table S7). Interestingly, two groups of differently expressed TFs with the highest number exhibited opposite dynamic expression trends from early to late stages: expression levels of 150 gene (cluster V, 24.75%) were highly expressed at prestage (stage01 and stage02), while in contrast, 72 genes in cluster VIII (11.88%) showed low expression at the prestage but highly expressed at the storage phases (stage03 and stage04). Some transcription factors related to meristem function and cell division such as HB (Homeobox), CPP, FAR1 were found in prestage (Figure 3B). Furthermore, 44.44% (4) of the ARFs (auxin response factors), responding to phytohormone auxin and important in determining plant architecture, were identified and their expression levels decreased gradually from early to late stages. This suggests that ARFs play important roles in early stages of barley grain development.

### 2.4. Prediction of RNA Editing Sites

**Figure 4:**
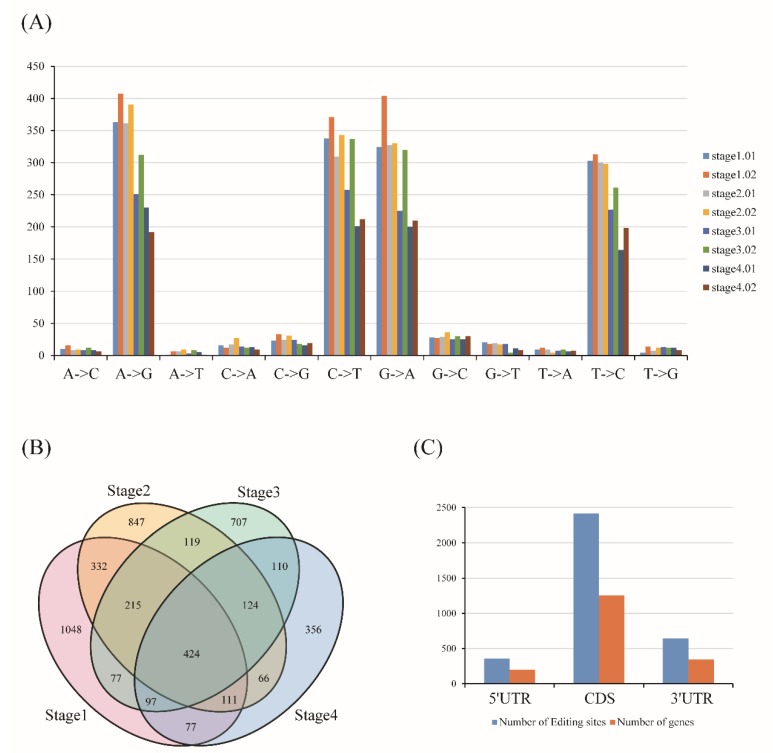
Identification of the RNA editing events during barley grain development. (**A**) The frequency of different types of nucleotide editing at four stages of barley grain development. (**B**) Venn diagram analysis of the abundance of RNA editing events among four stages of barley grain development. (**C**) The localization of RNA editing events in genic regions.

**Figure 5:**
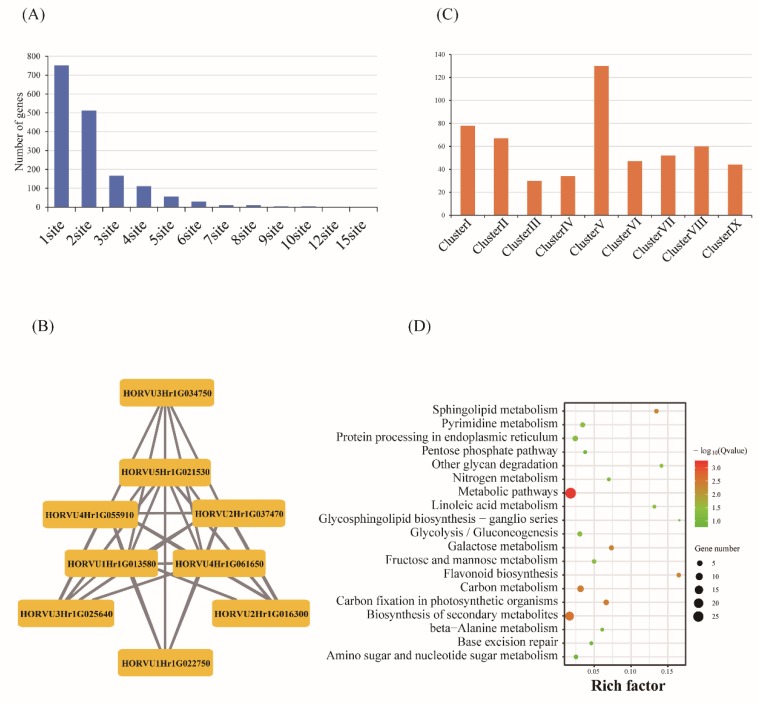
Frequency and functional enrichment of RNA-edited differentially expressed genes (DEGs). (**A**) The abundance of genes harbored the edit sites ranging from 1 to 15. (**B**) The regulatory network of the identified DEGs with RNA editing sites involved in embryonic defects. (**C**) Frequency of RNA-edited DEGs in nine expression clusters. (**D**) KEGG enrichment analysis of the RNA-edited DEGs identified in this study.

RNA editing is an important post-transcription mechanism to enrich genetic information and regulate diverse biological processes. After filtering the lower coverage sites as described in Method section, a total of 4711 editing sites were identified during the barley grain development, with 12 base change types, of which four editing types (A->G; C->T; G->A; T->C) were significantly higher than the other eight types (Figure 4A, Table S8). This result showed the transition occurred more abundantly than transversion in RNA editing, which was consistent with previous studies [23]. Then, the stage-specific and overlapped editing sites of these four stages were investigated. Results showed that stage01 owned the highest unique sites, with the number of 1048, followed by stage02, stage03, and stage04, with the numbers of 847, 707, and 356, respectively (Figure 4B). Among these editing events, a total of 3410 sites were found in 1795 protein-coding genes (Table S8), of which 2412 were found in CDS region, and 356 and 642 sites were located in 5′ and 3′ UTR regions (Figure 4C), respectively, suggesting that most editing could change the genetic information to result in the change of the encoded protein sequence. The gene HORVU7Hr1G000580 showed the most abundant editing sites with the total value of 15, and most of the other genes were observed to have one editing site (Figure 5A). Totally, 783 editing sites in 542 genes were observed to result in the amino acid change, of which 139 editing events (25.65%) led to a conversion from hydrophilic amino acid to hydrophobic amino acid, and 102 (18.82%) changing from hydrophobic amino acid to hydrophilic amino acid (Figure S6). A series of genes related to starch and sugar metabolic processes were found to be edited, such as beta-amylase 5 (HORVU4Hr1G089510), fructose-1,6-bisphosphatase class 1 (HORVU4Hr1G060630, HORVU1Hr1G074330) and low-molecular-weight glutenin subunit (HORVU1Hr1G001350, HORVU1Hr1G001420, HORVU1Hr1G001120, HORVU1Hr1G001120, HORVU1Hr1G000930, HORVU1Hr1G000930, HORVU1Hr1G001080) (Table S9). What is more, 4 terminators were found to be converted to 1 hydrophobic and 3 hydrophilic amino acids, and 1 hydrophobic and 11 hydrophilic amino acids were converted to terminators due to RNA editing. The embryo-defective related gene (HORVU2Hr1G037470) had 4 C to T editing events occur during barley grain development, and two of them caused the amino acid change (H>Y, A>V), one site with no amino acid change (C>C), and one resulted in the termination of the coding transformation (Q>*) (Figure S7). The stop code of this gene caused by editing may lead to the defect development of the embryo (Figure 5B). Furthermore, combined with the expression patterns, 308 DEGs were found to have RNA editing occur and the edited genes were more abundant in cluster I, cluster II, and cluster V, compared to cluster III, cluster IV, and cluster VII (Figure 5C). Then, KEGG analysis of them showed that they were enriched in metabolic pathways, biosynthesis of secondary metabolites, carbon fixation in photosynthetic organisms, carbon metabolism, base excision repair, pentose phosphate pathway, and glycolysis/gluconeogenesis (Figure 5D). These results demonstrated that RNA editing may function as the crucial regulator to control the complex process of barley grain development.

## 3. Discussion

### 3.1. mRNA Reveals Insights into Dynamic Regulation of Grain Development in Barley

Grain development represents an elaborate and complex phase in the plant life cycle and has been extensively studied in recent years [6,7,13]. It has been suggested that differences in the molecular regulation of grain development may arise from spatial and temporal variation in gene expression [1,2,18]. Hence, interpretation of transcriptomic data providing some clues for the development of grain in plants, and barley in particular, becomes imperative. In this study, we performed a comprehensive analysis of transcriptional dynamics during barley grain development using a transcriptomic sequencing strategy. By pairwise comparisons of the data between four stages, differential transcript abundance and sequence variation were found. The levels of transcripts showed close correlation with the physiological changes during grain development. The majority of the genes exhibited peak expression at the prestorage phase (stage01 and stage02), and relatively low expression at the storage and mature phases (stage03 and stage04). We demonstrated that a dramatic change took place during the transition of the seeds from a prestorage phase to maturation, which enhanced our understanding of the developmental shifts during the filling process of barley grain. Previous studies have identified several pathways or genes involved in regulating barley grain development based on microarray hybridization [1,7,12,14]. In contrast to cDNA chip hybridization, the RNA-seq technology has obvious advantages for transcriptome dynamics analysis, which could generate millions of sequence reads with high reproducibility, especially for the lowly expressed transcripts. In the current study, 48.23% (19,165/39,734) of the annotated barley genes were detected to have the RPKM value more than 1 in at least one stage. These results were similar with that of hexaploid wheat (~55%) [2] and Arabidopsis (~44%) [18], but significantly higher than previous studies in barley [1,6,7,12,14]. Comparison analysis revealed that a total of 9527 genes were considered to be potentially involved in the regulation of barley grain development. These genes were significantly enriched in 16 KEGG pathways (Figure S8A), which provided the clues to explore the global activation of metabolic pathways and gene regulatory networks activated during barley seed development.

### 3.2. Identification of Genes Required for Grain Development in Barley

Seed development is a complex process in which most of the essential developmental processes are initiated, including embryo and endosperm development, storage essence accumulation, desiccation tolerance forms, and seed dormancy. To obtain the information concerning the genes governing this complex developmental process, several studies have been focused on the identification of “essential” genes (lethal-phenotype genes) for seed development [3,9]. At present, a total of 481 genes were characterized as “essential” genes required for normal embryo development in Arabidopsis and presented in the SeedGenes database (http://seedgenes.org/index.html). The majority (74.43%) of these genes have been confirmed by function analysis [9]. In this study, we identified 3172 seed development-related genes with significant similarity to Arabidopsis based on bidirectional best BLAST hits and an e-value threshold below 1e-30. Among them, 887 genes were significantly differentially expressed during barley grain development and could be considered as potential barley EMB genes (Table S10). Mutations of the majority of these genes (85.34%, 757/887) will cause defects in embryo development. While the other 100 EMB genes are essential to seed pigmentation, mutation of these genes will lead to morphologically normal seeds with color changes such as albino and pale green [3,9]. To define their detailed functional categories, KEGG enrichment analysis of the potential EMB genes for barley used KOBAS (version 3.0) software [24]. Enrichment analysis found that the nucleotide, protein, lipid, and carbohydrate metabolism pathways were significantly enriched (Figure S8B). While these results demonstrate that the majority of genes related to seed development of Arabidopsis [3,9] are also present in barley grains, the definitive functions of these genes in barley seed development remain to be determined.

Developing barley grains are green and contain functional chloroplasts capable of photosynthesis during seed filling in barley. The prestorage phase (0–5 DAP) and transition phase (6–10 DAP) are characterized mainly by cell division and absence (or initial accumulation) of starch in the endosperm [6]. Previous studies in Arabidopsis indicated that the level of photosynthesis was high in the early and middle stages of grain development, when the majority of photosynthesis-related genes showed peak expression [5]. Our findings showed that a total of 29 genes were clustered as photosynthesis-related genes and 68.97% (20/29) of them peaked at stage02, represented by photosynthesis, photosystem I, and photosystem II. For example, several genes encoding photosynthetic light reaction (such as light-harvesting complex II, including Lhca1, Lhca2, Lhca3, Lhca4, Lhcb1, Lhcb2, Lhcb3, Lhcb5, Lhcb6, and Lhcb7) were low in the youngest seeds, increased sharply by 8 DAP (stage02), and then decreased afterwards.

### 3.3. Plant Hormone Signal Transduction and Sugar Signaling Interaction Networks During the Transition from Pre-Storage to Storage Phase

Plant hormone plays a critical role in the entire growth period in plants. For instance, auxin contributes mainly to cell enlargement and division, and ABA (Abscisic acid) is believed to be involved in the initiation of storage compounds synthesis and linked to seed desiccation tolerance level and seed dormancy [1,4,5,7,14,25,26]. The plant hormone signal transduction pathway bridges the hormone and its response genes. Our transcriptome analysis indicated that auxin signal transduction-related genes were related to cell enlargement and division and their expression showed peak at prestore stage (Figure S9A), but the ABA signal transduction pathway showed a peak at store stage (Figure S9B).

Extensive studies have elucidated that ABA has an important role in the genetic control of seed development. There are four TFs known as main regulators of late embryogenesis in A. thaliana. These regulators are ABA-dependent genes, including LEC1 (HAP3 subunit), LEC2 (B3-domain), ABI3 (ABA-insensitive 3), and FUSCA/FUS3 (fused cotyledon3) [4,14,26]. FUS3 regulates endogenous ABA synthesis, which will activate the ABA-insensitive protein phosphatases 2C (PP2C) and sucrose nonfermenting 1 (Snf1)-related protein kinases 2 (SnRK2). SnRK2 subsequently initiates downstream targets, including ABF, AREB, and ABI5 [4,26]. In this study, we found several ABA signal transduction-related genes were differentially expressed in barley during grain development, and HvPP2C, HvSnRK, and HvABI were preferentially expressed at the storage phase (Figure S9B).

It has been demonstrated that both sugars and ABA played critical roles in controlling the transition from the cell division phase to the cell enlargement phase as storage reserves were accumulated [4,26]. SnRK, known for the regulation of key starch biosynthesis genes, such as sucrose synthase (SUS) and ADP-glucose pyrophosphorylase (AGPase), has been shown to act as a bridge between ABA and sugar signaling [4,26]. During legume seed development, glucose and sucrose acted in almost opposite fashions, with glucose promoting cell division and sucrose being associated with cell expansion [4]. Importantly, previous studies regarding metabolite contents in developing barley endosperm showed that hexoses were highest during early development whereas sucrose peaked at 8 DPA [13,16]. In the current study, our data showed some sugar-related genes were significantly expressed to maintain different contents of glucose and sucrose in distinct stages, thus medicating the phase transition from prestorage to storage phase. In potato tubers, for instance, the expression of invertase or sucrose phosphorylase bypasses sucrose synthase and decreases sucrose levels [4]. Two genes encoding sucrose phosphorylase were significantly expressed in barley and all of them showed a peak expression level at the prestorage phase (stage01 and stage02). It appears that the critical role these sugar-related genes played was to maintain a relatively low content of sucrose at the early stages (Figure S9C). In addition, one sucrose synthase gene, together with an enzyme that hydrolyzes sucrose to glucose and fructose, was preferentially expressed at the early stage, contributing to the high content of glucose at this stage [14] (Figure S9C). Starch biosynthesis was detected at high levels at the storage phase (stage03, stage04) (Figure S9D). A similar pattern was found for one sucrose phosphate and two sucrose synthases genes, indicating their important roles in starch biosynthesis at the storage phase. To conclude, we might hypothesize that the extensive interactions between sugar and ABA signaling have critical roles in mediating the phase transition of barley grain development according to the results obtained in this study.

### 3.4. RNA Editing Regulated Barley Grain Development

RNA editing is an important post-transcription mechanism to enrich genetic information and regulate diverse biological processes by altering the genetic identity between RNA and genomic DNA. Although the RNA editing also occurs in the noncoding regions, such as 3′-UTR and 5′-UTR, rRNA and tRNAs, it mostly occurs in protein-coding regions, resulting in the change of encoded protein as well as protein function [27,28,29]. A series of studies have evidenced that the RNA-editing events have important roles in regulating organ formation, architecture growth, and development [30,31,32,33]. In this study, during grain development, the editing DEGS were more observed in the prestore stages (cluster V) than store stages (cluster VII) (Figure 5C). In addition, RNA editing was found to occur in some of the genes involved in the embryonic defects development and breakdown of the macromolecule organic matter, and editing has caused the stop coding or amino acid change (Figure S7; Tables S8 and S9), consistent with the empirical evidence that mostly nucleic acid activity and cell cycle occur in embryonic development stages. Combined with the DEGs, KEGG pathway enrichment analysis showed that the editing genes were significantly enriched in metabolic pathways, carbon fixation in photosynthetic organisms, base excision repair, and glycolysis/gluconeogenesis, which is consistent with the physiological process of spike development (Figure 5D). Thus, the RNA editing may function as a ‘regulator’ to ensure the plant’s normal growth and development.

## 4. Materials and Methods

### 4.1. Sample Preparation and Sequencing Library Construction

Barley plants (cv. Clipper) were grown in field trial of Northwest A&F University (Yangling, Shaanxi, China) under normal agricultural management. The time of anthesis and developmental stage of caryopses were determined for each head based on the dissection of the middle spikelet as previously described [13]. Individual spikes were tagged at the time of anthesis and harvested in the morning (08:30–09:30 h) at 3, 8, 13, and 18 DPA [16]. Four biological replicates were sampled. For each sample, tissues were collected directly into liquid nitrogen by pooling the middle parts of the spike from 10 to 15 individual plants for the purposes of homogeneity. 

Then, two of the four replicates were randomly selected to isolate total RNAs using TRIzol reagent (Invitrogen, Waltham, MA, USA) and were treated with RNase-free DNase I to remove any contaminating genomic DNA. The purified RNAs were used to construct the RNA sequencing library following the Illumina’s standard pipeline (Illumina, San Diego, CA, USA). In brief, mRNAs were extracted from purified RNA by Dynabeads oligo (dT) (Dynal, Invitrogen). Double-stranded cDNAs were synthesized using reverse transcriptase (Superscript II, Invitrogen) and random hexamer primers. After size selection and purification through agarose gel electrophoresis, the selected fragments with the length of 500 bp were ligated to sequencing adaptors. Then, the ligated RNAs were reverse transcribed and amplified for Illumina sequencing. The high-throughput sequencing was performed by pair-end (PE) approach with the read length of 100 bp on Illumina HiSeq2000 platform following the standard protocol at BGI Comp (Shenzhen, China). Each sample was sequenced with no less than 60 million reads. All the high-throughput sequencing data were deposited into the Sequence Read Archive (SRA) database in NCBI with the accession number of SUB2126402.

### 4.2. Expression Profiles Analysis

After removing the adaptor sequences, empty reads, and low-quality reads (Q < 20) with FASTX-toolkit (FASTX-toolkit 0.0.14, http://hannonlab.cshl.edu/fastx_toolkit/), all the clean reads were aligned to barley genome [17] using the default parameters for HISAT2 (version 2.0.5) [34]. Reads counts statistic was calculated using HTSeq (Version 0.7.1) [35] with htseq-count command. Then, gene expression levels were estimated using RPKM values (Reads Per Kilobase transcriptome per million reads). Each sample was treated individually. Pearson correlations between biological replicates were conducted based on the RPKM values of all expressed genes using the function cor in R, which were further used to generate a dendrogram of samples.

### 4.3. Identification of Differentially Expressed Genes (DEGs)

Differential expression analysis was performed using R packages of DESeq2 (version 1.20.0) between any two stages with two biological replicates [8]. Genes with an adjusted *p*-value < 0.05 and at least twofold changes (|Log2 (treatment/control)| ≥ 1) were considered as differentially expressed. Then, a K-means clustering was used to extract the fundamental patterns of gene expression inherent in the development of barley caryopses [36]. The following three additional steps were performed to pretreat the dataset using the standard tools in cluster 1.52 [37]: (i) Log-transform the data and all data values (FPKM) were replaced by log2(FPKM); (ii) for center arrays [mean], subtract the row-wise mean from the values in each row of data; (iii) normalize arrays: specifies to normalize each row in the data so that the sum of the squares of the values in each row is 1.0. The Venn diagrams in this study were prepared using the Vennerable package (version 3.0) in R based on the gene list for each tissue type.

### 4.4. Identification of the Transcript Factors in DEGs

All the nucleotide sequences for DEGs were extracted from the barley genome [17] and then used for prediction of the potential transcript factors by the iTAK (version 17.09) online software [38].

### 4.5. RNA Editing Analysis

All potential RNA editing sites were predicted using SPRINT (SnP-free Rna editing IdeNtification Toolkit) [39] software with default parameters. To support the prediction, we also identified the SNP between RNA reads and reference genomic DNA using SNP calling tools Samtools (version 1.3.1) (http://www.htslib.org) to analyze the mapping BAM files. The overlapped results obtained by both methods remained for further analysis. Then, the editing sites were further filtered using the following parameters: (1) the edited sites had more than five mapped reads; (2) the ratio of editing reads and total mapped reads more than 50%; (3) the editing sites were found in both biological replications.

### 4.6. Gene Ontology (GO) and KEGG Enrichment Analysis

GO terms that are significantly overrepresented in each cluster were determined by the AgriGO (version 2.0) [40]. Singular Enrichment Analysis (SEA) in AgriGO was used to detect over-represented GO categories in each cluster compared to the whole genes. GO terms with corrected Padj less than 0.05 were taken as significant ones. KEGG was used to identify processes and pathways of specific gene sets [41]. The annotations on genes were carried out using a simple unidirectional BLAST (blastx) search against already classified proteins from Arabidopsis thaliana (thale cress), Oryza sativa japonica (Japanese rice) (RefSeq), and Zea mays (maize) by KAAS (KEGG Automatic Annotation Server) software [41]. Then, KOBAS (version 3.0) software was used to enrich the pathways of all the genes [24].

## 5. Conclusions

In this study, we systematically investigated the gene expression atlas and RNA editome during barley grain development. Results showed that the expression profiles of transcripts involved in both primary and secondary metabolism were significantly changed between the transitions from prestorage phase to storage phase, which could reflect the physiological changes of grain development. Furthermore, the essential gene and regulatory networks involved in this process were investigated. Results showed that plant hormone signal transduction and sugar signaling interaction networks could play a crucial role in regulating grain development. Finally, 4771 RNA editing sites were detected in these four development stages, and most RNA editing genes were preferentially expressed at the prestore stage compared to store stage, suggesting that RNA editing might act as a ‘regulator’ to control grain development. This is the first study to report RNA edit events involved in grain development in barley at the whole transcriptome level. Our study systematically dissected the transcriptional dynamics of barley grain development, which will contribute to improved understanding of the molecular mechanism and dynamics of gene regulation underlying grain development in barley and beyond.

## Supplementary Materials

Supplementary materials can be found at https://www.mdpi.com/1422-0067/20/4/962/s1; Figure S1: The photos of four stages during barley grain development; Figure S2: RNA-seq read mapping counts against the barley reference genome; Figure S3: The correlation relationship of the gene expression level between four stages reported in this study and two stages in barley RNA-seq database; Figure S4: The volcano map of the number of differently expressed genes between two adjacent stages in barley grain development. A: stage 1 vs. stage 2; B: stage 2 vs. stage 3; C: stage 3 vs. stage; Figure S5: Clusters analysis of genes showing representative expression patterns during barley grain development; Figure S6: The abundance of change type among hydrophobic and hydrophilic amino acid as well as terminator caused by RNA editing; Figure S7: The aligment of the normal and edited protein sequences of the embryonic defect-related gene HORVU2Hr1G037470; Figure S8: Functional characterization of the differentially expressed genes during barley grain development; Figure S9: Identification of the DEGs involved in hormone signal transduction and sugar metabolism processes; Table S1: RNA-seq read mapping counts against the barley reference genome; Table S2: The expression of genes of all the stages(FPKM>=1); Table S3: The GO enrichment of pre-store stage genes; Table S4: The GO enrichment of store stage genes; Table S5: The set of genes showing stage-specific expression during barley grain development (FPKM < 1 in all other stages); Table S6: Go enrichment of the DEGs clustered in different clusters; Table S7: The detail list of transcription factors (TFs) differentially expressed during barley grain development; Table S8: RNA editing sites in coding regions of morex genome predicted by SPRINT tool; Table S9: The function annotation and expression level of editing DEGs; Table S10: List of putative EMB genes for barley grain development.
