# Supplementary material for: Transcriptional Dynamics of Grain Development in Barley (Hordeum vulgare L.)
Source: Int J Mol Sci. 2019 Feb 22;20(4):962. doi: 10.3390/ijms20040962 (PMC6412674; doi:10.3390/ijms20040962)
Supplement: Supplementary file 1 [file ijms-20-00962-s001.zip › Supplementary Figures.docx]

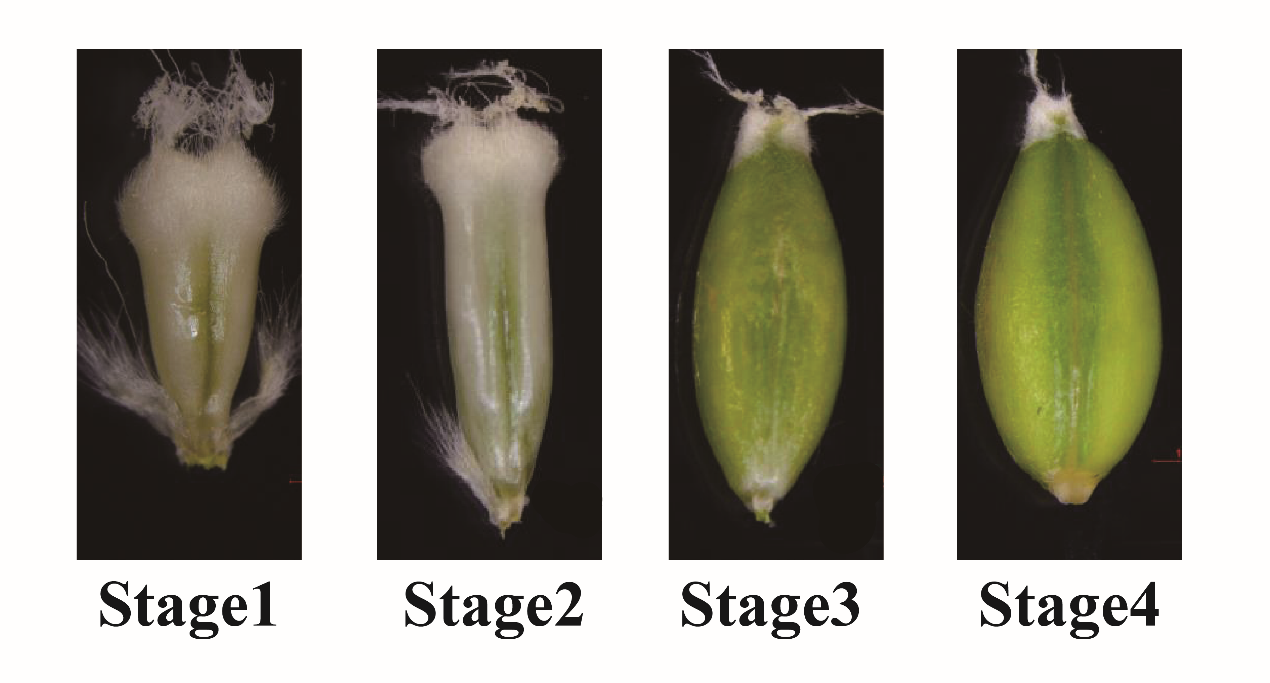


**Fig. S1**. The photos of four stages during barley grain development. Stage1: early pre-storage phase (3 days post-anthesis (DPA)); Stage 2: late pre-storage or transition phase (8 DPA); Stage 3: early storage phase (13 DPA); Stage 4: levels off stages (18 DPA).


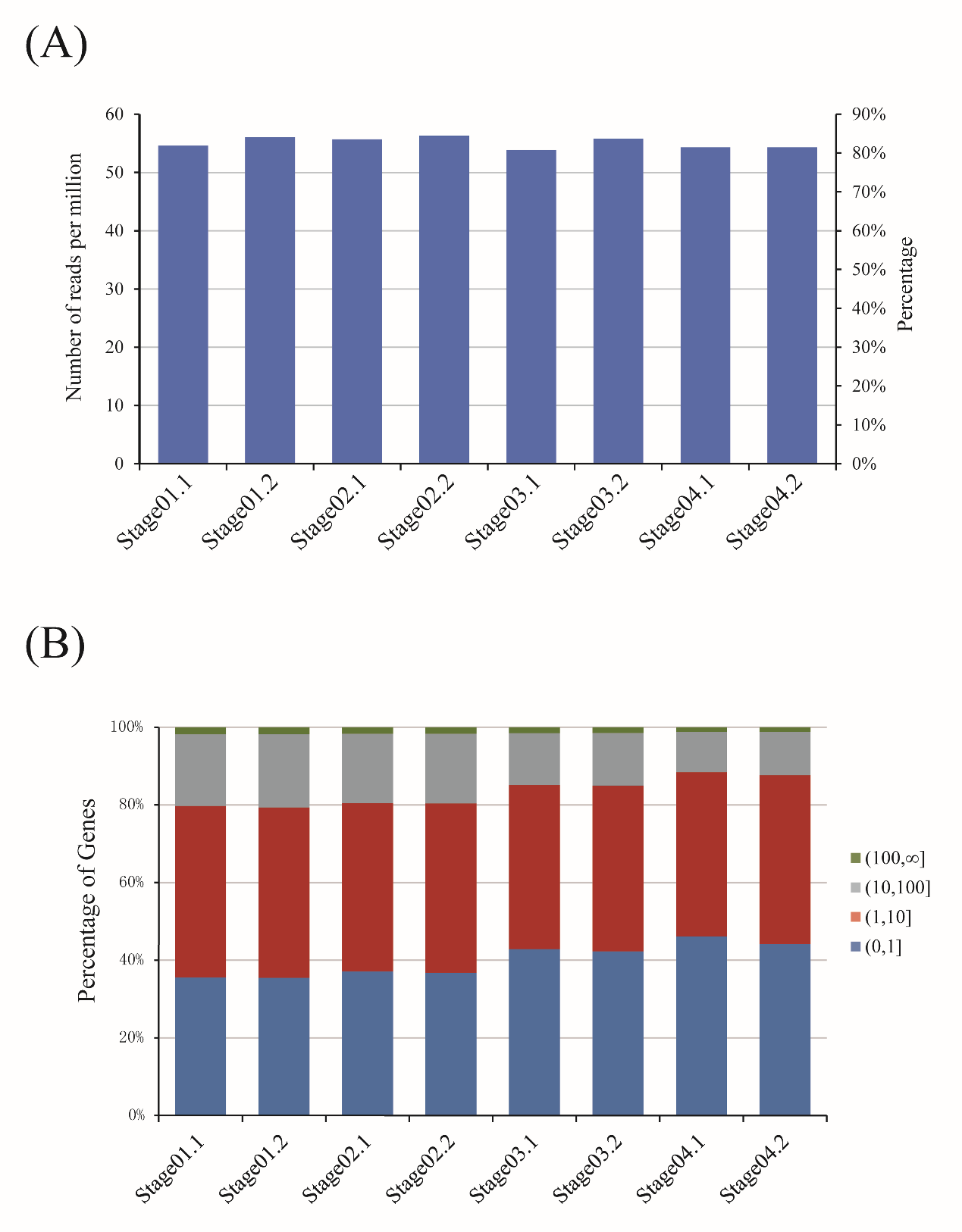


**Fig. S2.** RNA-seq read mapping counts against the barley reference genome. **(A)** RNA-seq read mapping counts against the barley reference genome. **(B)** Proportions of genes with different expression levels in barley. The different colors represent the value of FPKM.

**
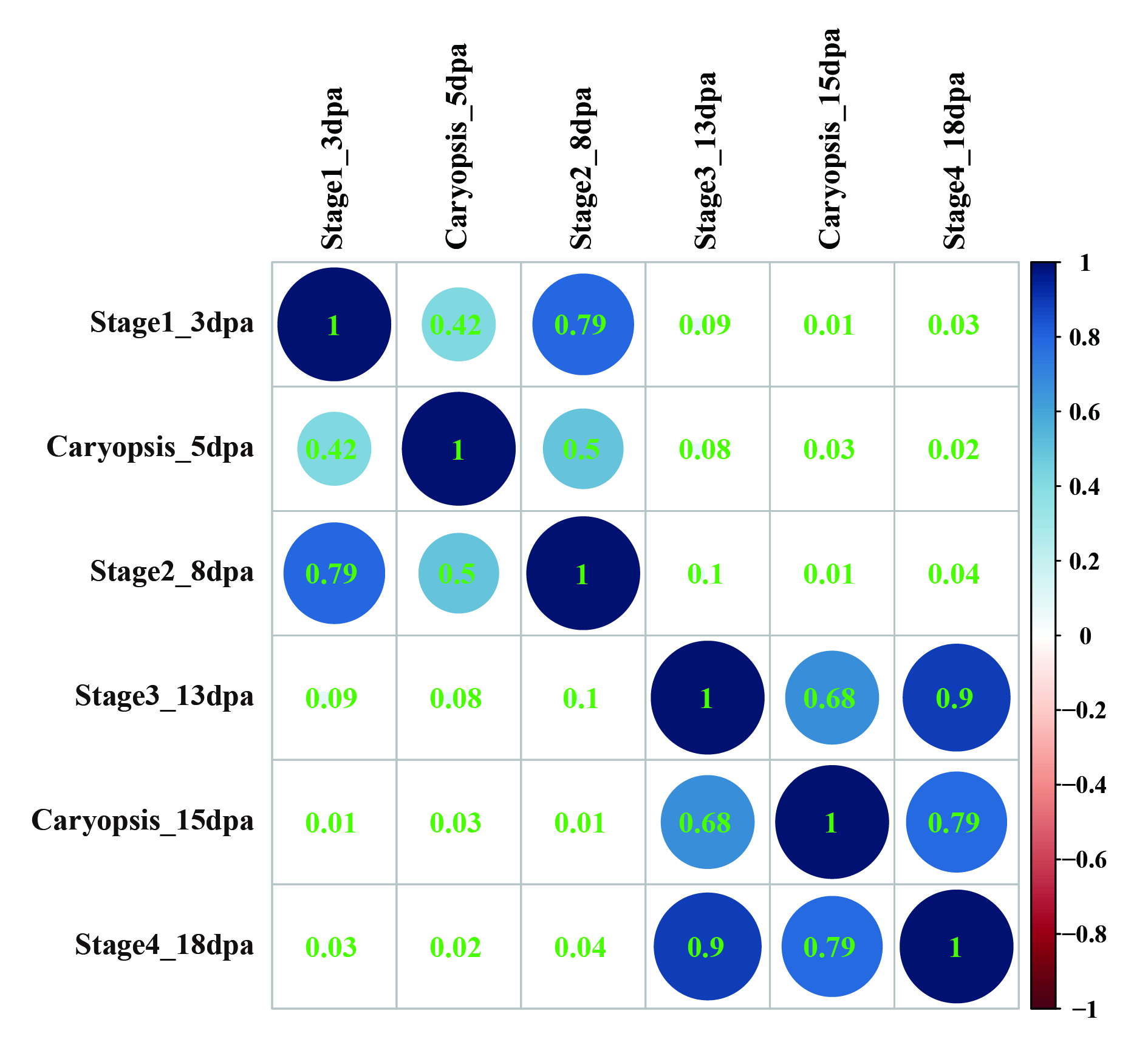
**

**Fig. S3**. The correlation relationship of the gene expression level between four stages reported in this study and two stages in barley RNA-seq database.


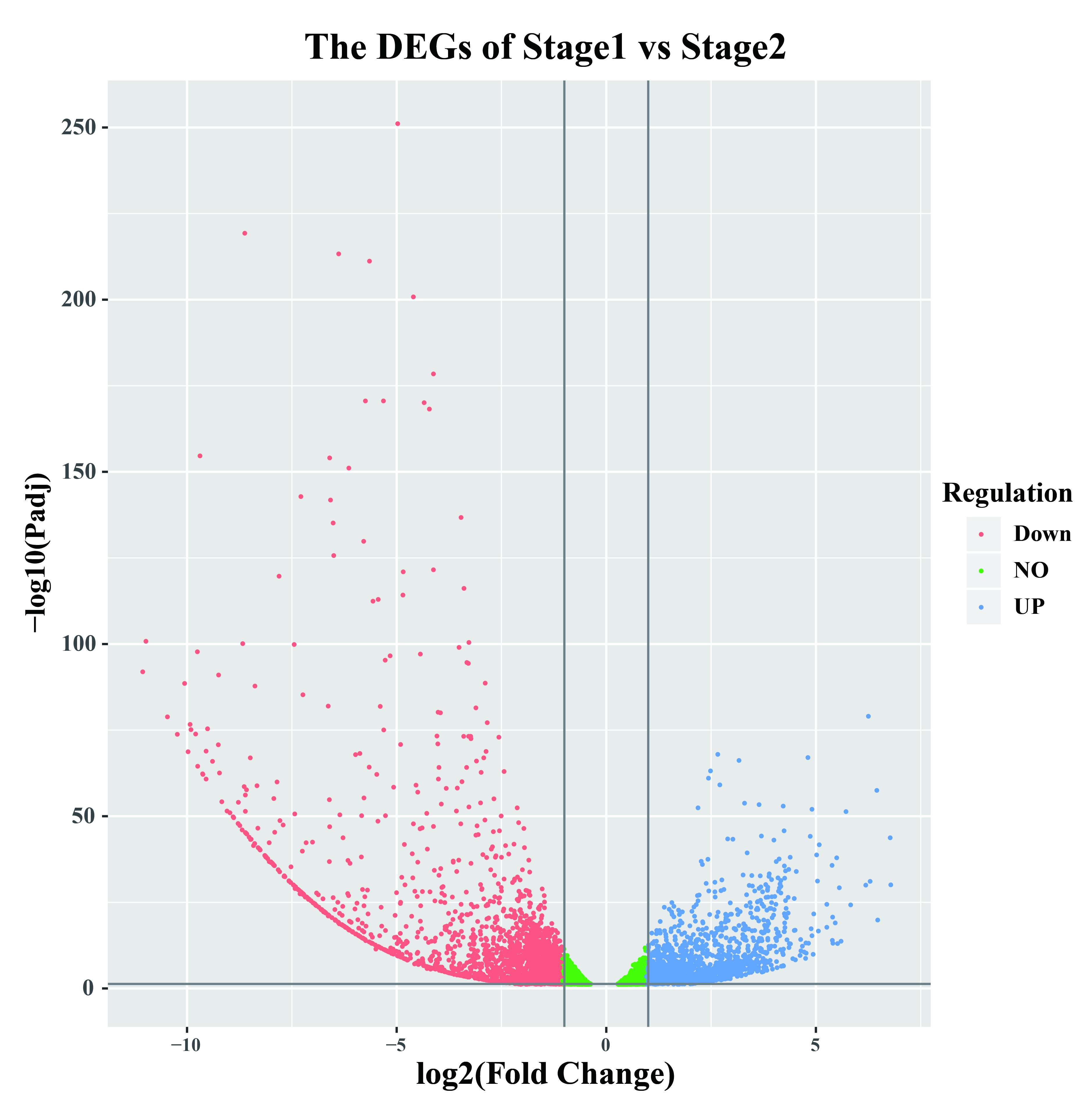

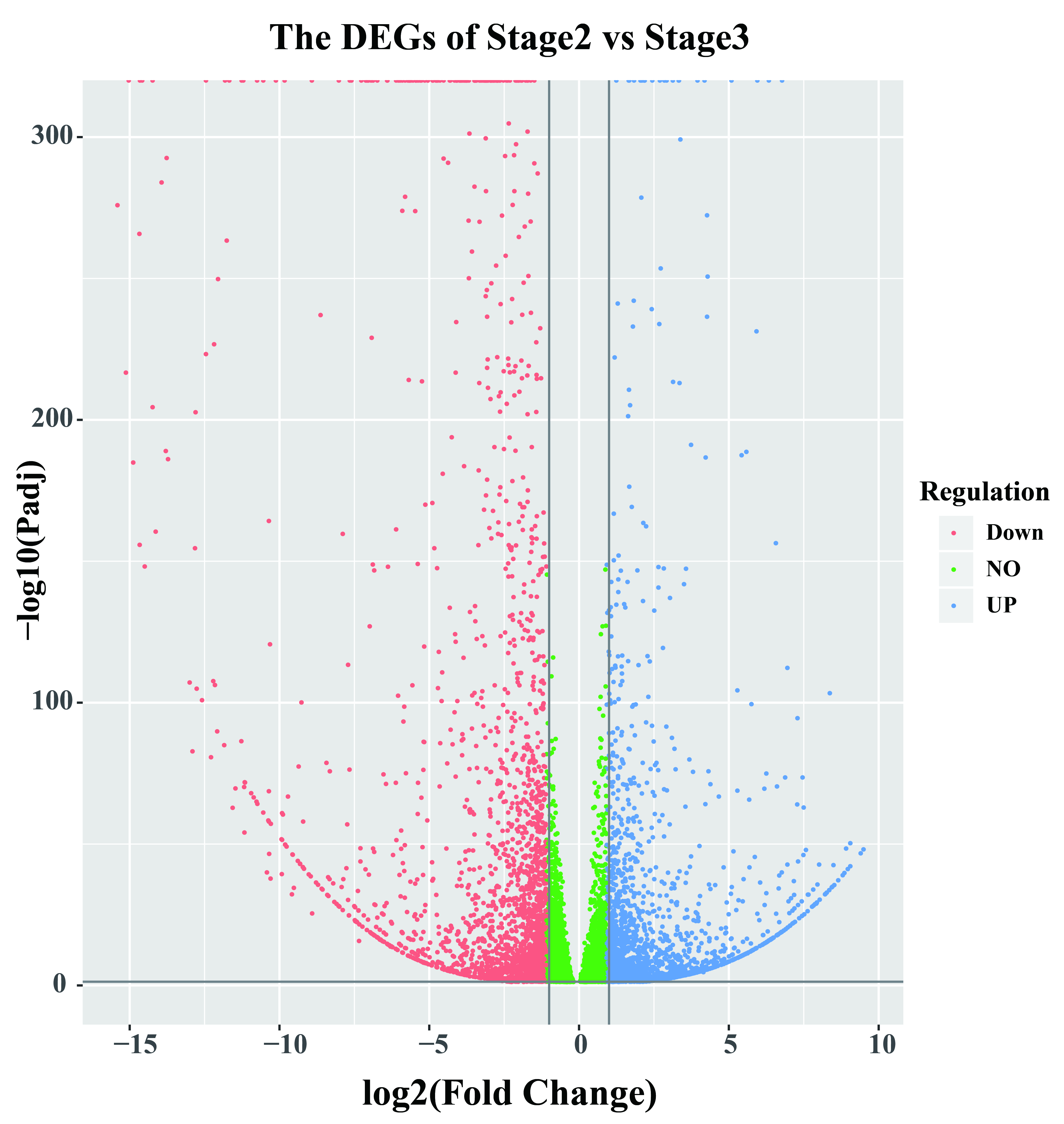

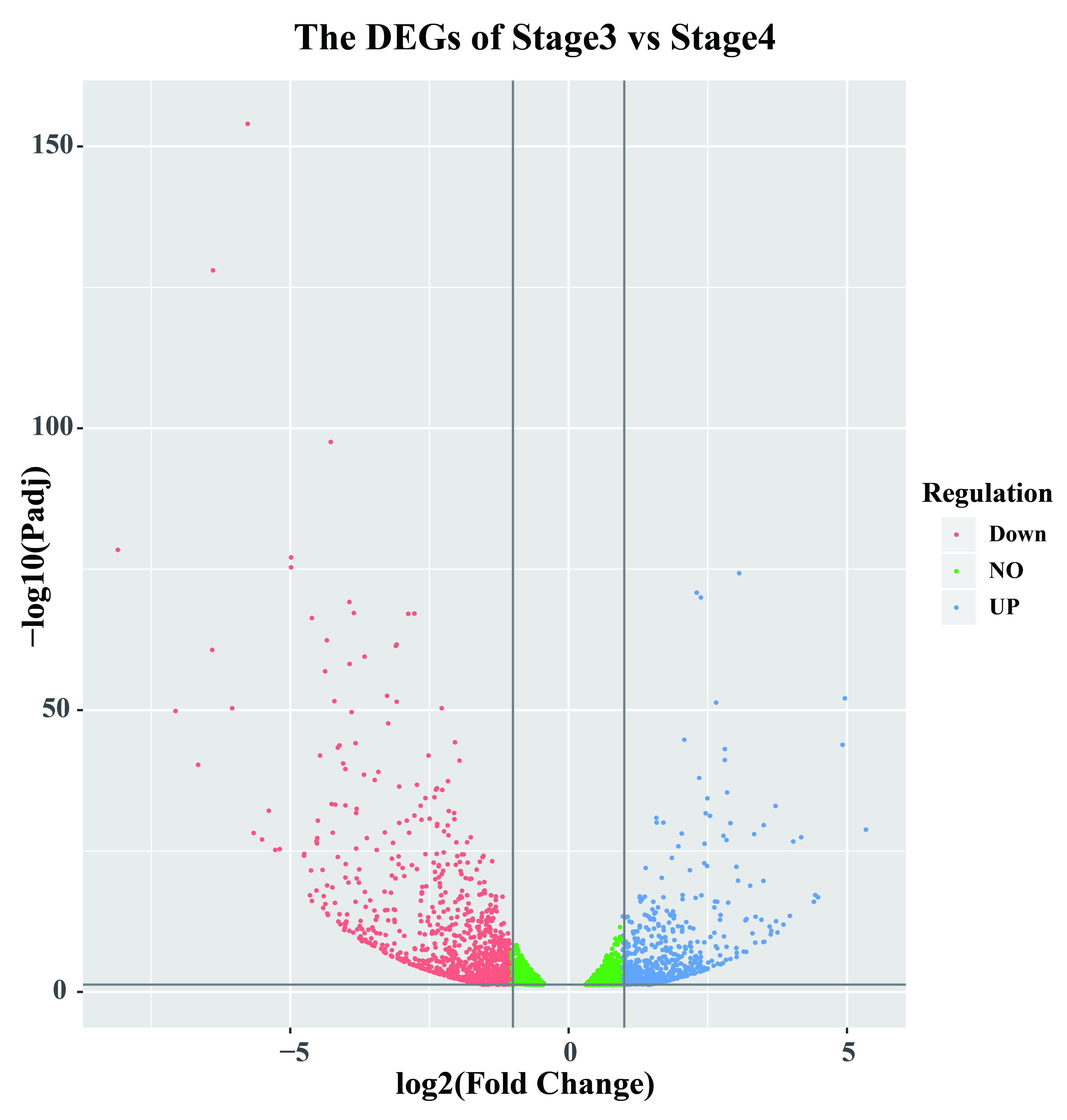


B

C

A

**Fig. S4**. The volcano map of the number of differently expressed genes between two adjacent stages in barley grain development. A: stage 1 *vs* stage 2; B: stage 2 *vs* stage 3; C: stage 3 *vs* stage 4


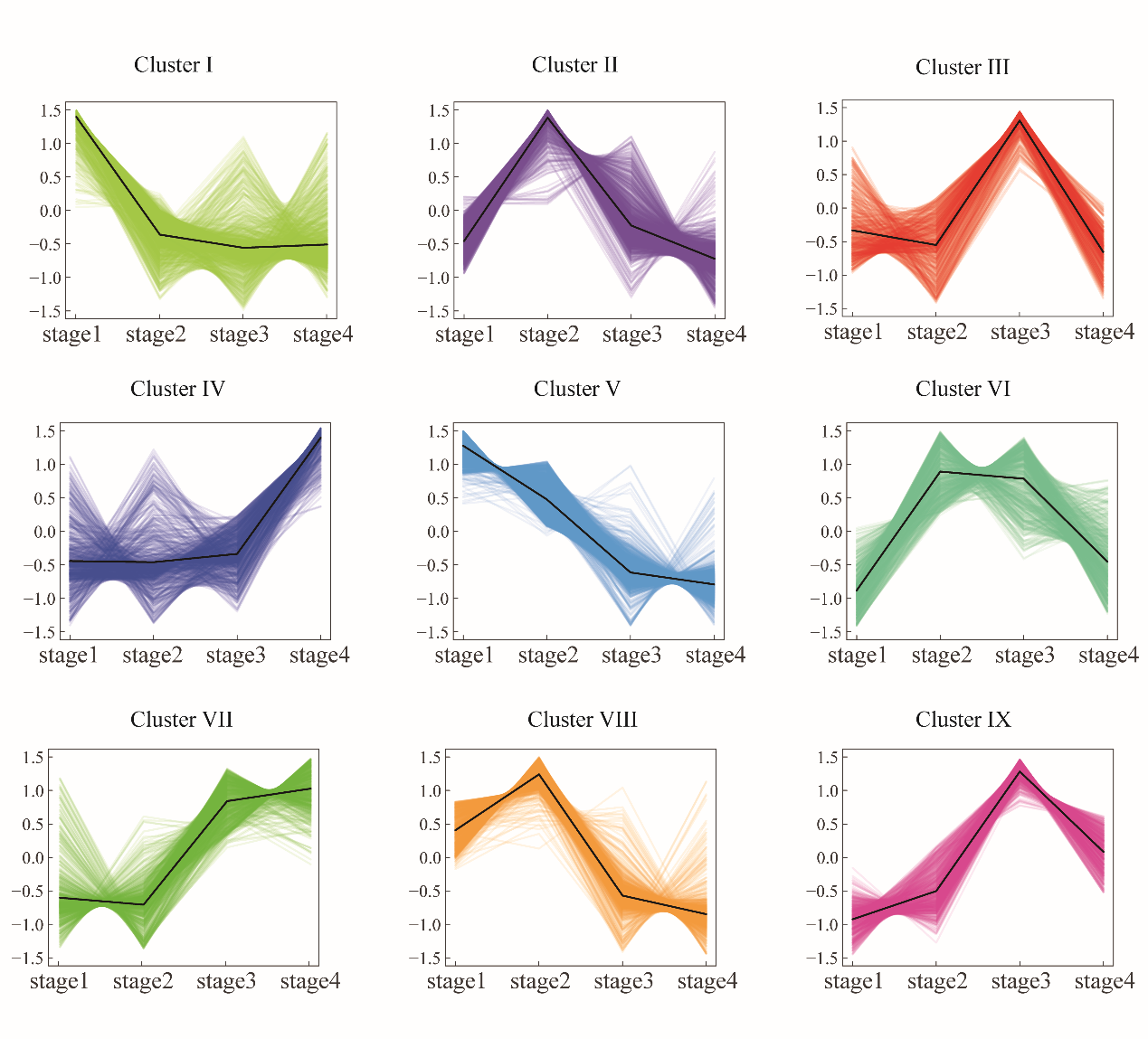


**Fig. S5.** Clusters analysis of genes showing representative expression patterns during barley grain development.


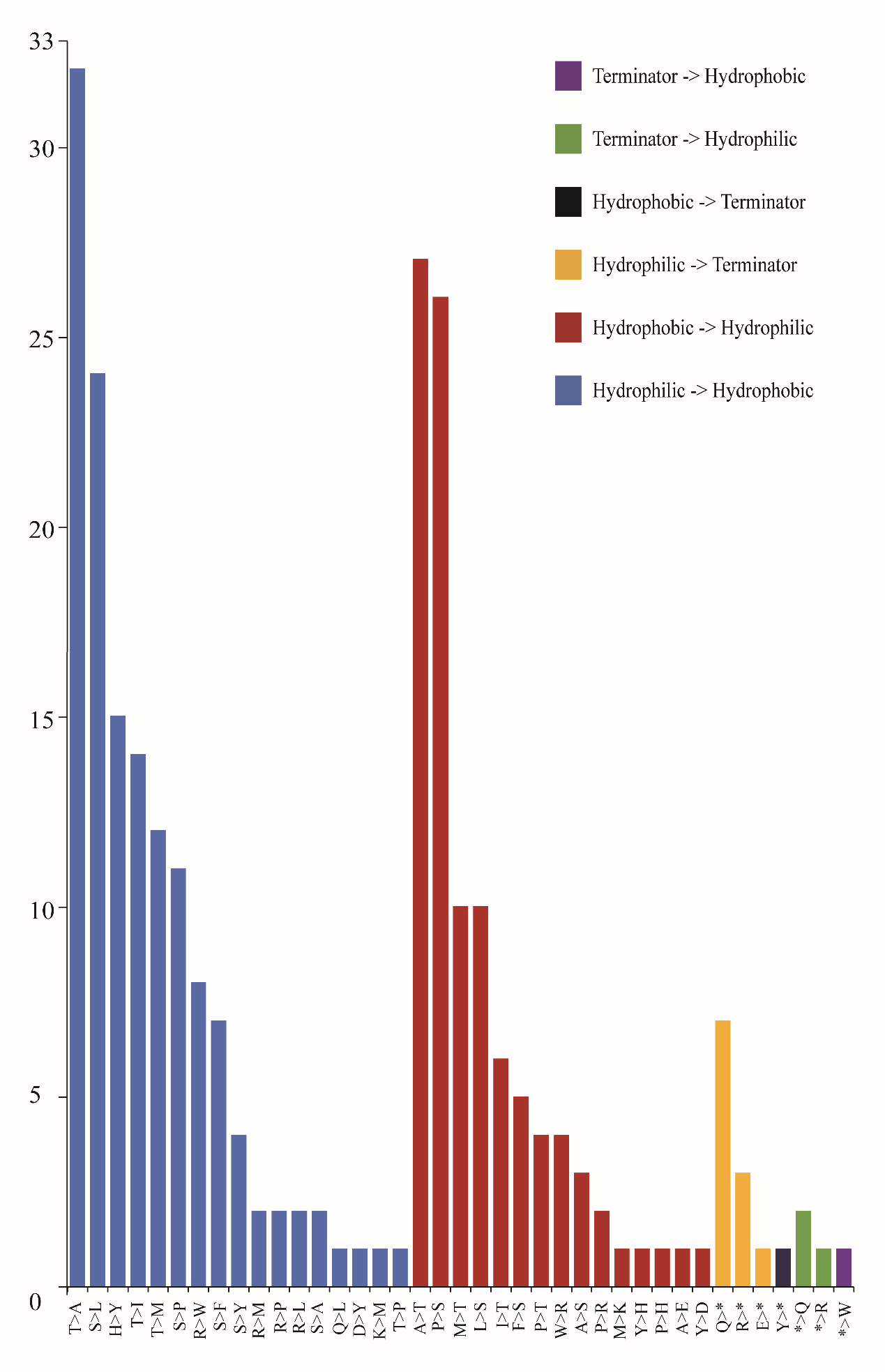


**Fig. S6.** The abundance of change type among hydrophobic and hydrophilic amino acid as well as terminator caused by RNA editing.


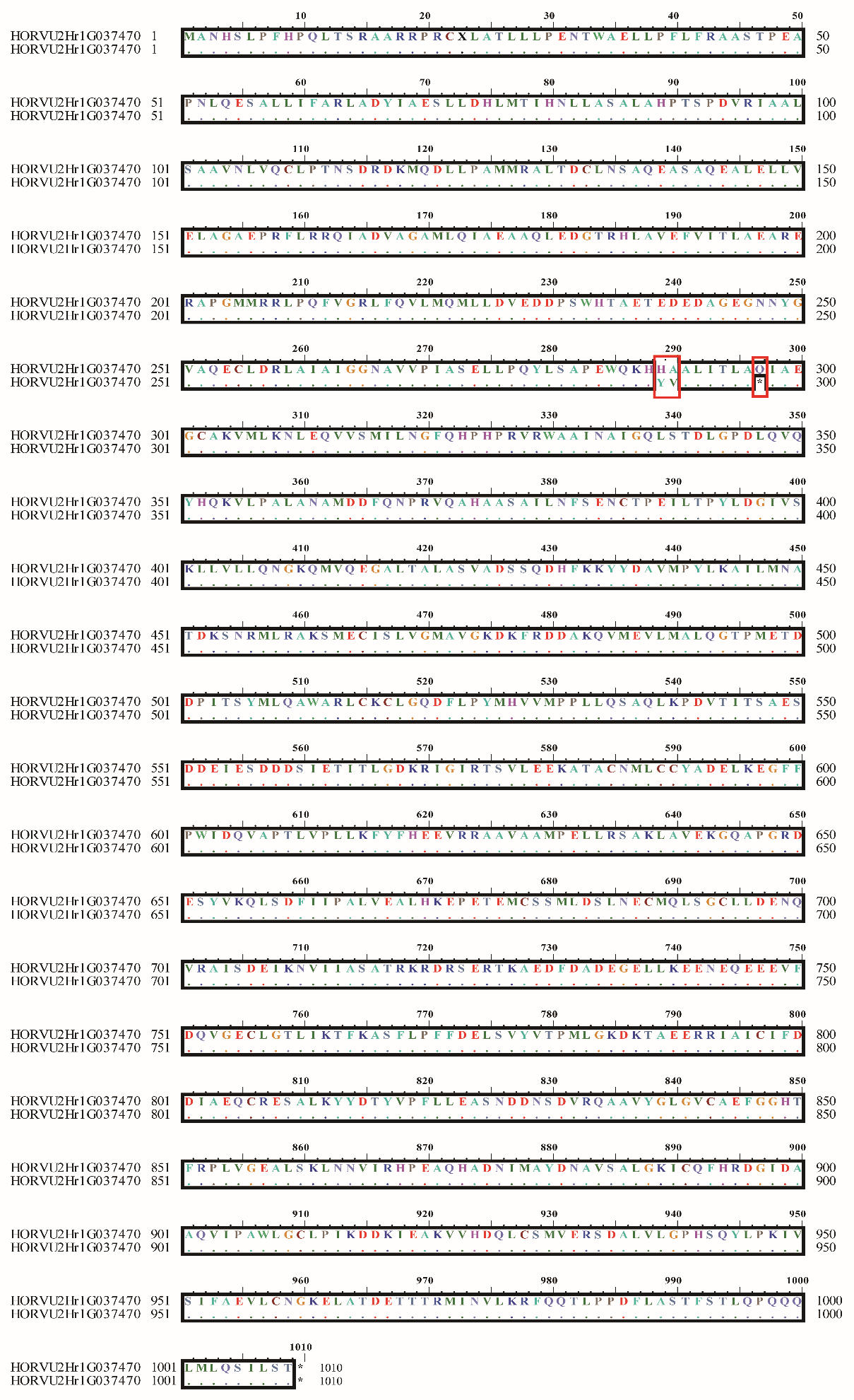


**Fig. S7**. The aligment of the normal and edited protein sequences of the embryonic defect-related gene HORVU2Hr1G037470.


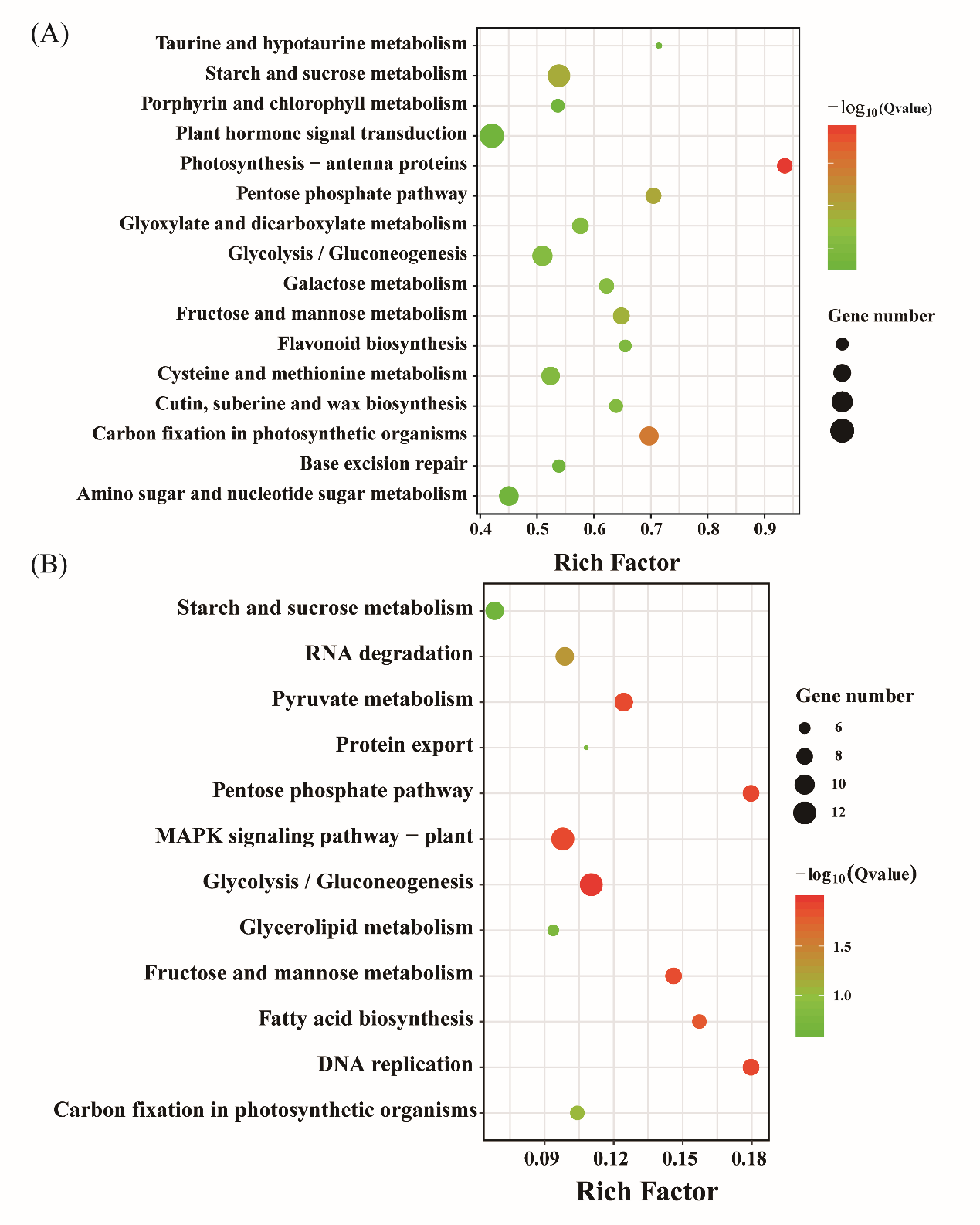


**Fig. S8.** Functional characterization of the differentially expressed genes during barley grain development. (A) KEGG enrichment analysis of all of the DEGs. (B) KEGG enrichment analysis of the potential barley EMB genes.


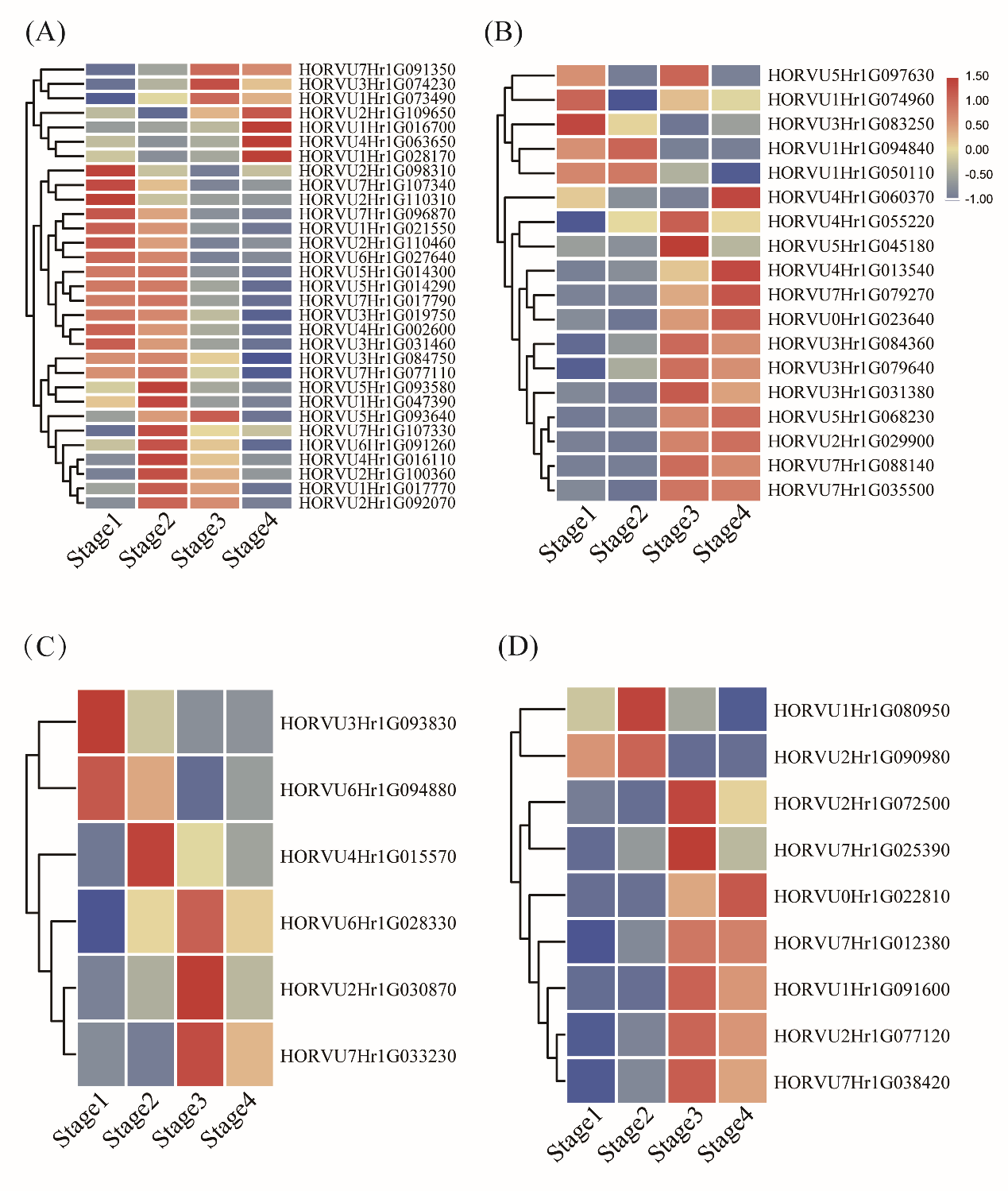


**Fig. S9.** Identification of the DEGs involved in hormone signal transduction and sugar metabolism processes. (A) Heatmap of the expression level of the auxin signal transduction related DEGs in four stages. (B) Heatmap of the expression level of the DEGs involved in ABA signal transduction related in four stages. (C) Heatmap of the expression level of the sucrose metabolism related DEGs in four stages. (D) Heatmap of the expression level of the starch metabolism related DEGs in four stages.
